# Direct Estimation of Electric Field Distribution in Circular ECT Sensors Using Graph Convolutional Networks

**DOI:** 10.3390/s25206371

**Published:** 2025-10-15

**Authors:** Robert Banasiak, Zofia Stawska, Anna Fabijańska

**Affiliations:** Faculty of Electrical, Electronic, Computer and Control Engineering, Institute of Applied Computer Science, Lodz University of Technology, 90-924 Łódź, Poland; zofia.stawska@p.lodz.pl (Z.S.); anna.fabijanska@p.lodz.pl (A.F.)

**Keywords:** electrical capacitance tomography, graph neural networks, forward problem approximation, electric field prediction

## Abstract

The Electrical Capacitance Tomography (ECT) imaging pipeline relies on accurate estimation of electric field distributions to compute electrode capacitances and reconstruct permittivity maps. Traditional ECT forward model methods based on the Finite Element Method (FEM) offer high accuracy but are computationally intensive, limiting their use in real-time applications. In this proof-of-concept study, we investigate the use of Graph Convolutional Networks (GCNs) for direct, one-step prediction of electric field distributions associated with a circular ECT sensor numerical model. The network is trained on FEM-simulated data and outputs of full 2D electric field maps for all excitation patterns. To evaluate physical fidelity, we compute capacitance matrices using both GCN-predicted and FEM-based fields. Our results show strong agreement in both direct field prediction and derived quantities, demonstrating the feasibility of replacing traditional solvers with fast, learned approximators. This approach has significant implications for further real-time ECT imaging and control applications.

## 1. Introduction

Electrical Capacitance Tomography (ECT) is a non-invasive imaging technique widely used for monitoring multiphase flow, process control, and material characterization in industrial environments [1,2]. It reconstructs the permittivity distribution inside a domain based on capacitance measurements between multiple electrodes positioned along the domain boundary [3]. The forward problem in ECT consists of solving for the electric field distributions generated by each electrode excitation pattern, from which an independent set of capacitances is derived. The fields also play a further role in determining the sensitivity model of the system and, consequently, its image reconstruction performance.

Traditionally, the ECT forward problem is addressed using the Finite Element Method (FEM), which provides high-accuracy solutions for the electric field and potential distributions in complex geometries. However, FEM solvers are computationally intensive and time-consuming, making them unsuitable for high-throughput or real-time imaging tasks. As ECT applications increasingly demand faster acquisition and processing, there is growing interest in surrogate models that can approximate the forward model with significantly reduced computational cost.

Recent advancements in machine learning, particularly Graph Convolutional Networks (GCNs), a specialized form of Convolutional Neural Networks (CNNs), have shown promise for modeling spatially distributed physical phenomena [4,5]. While CNNs are well suited to learning mappings from structured input data, such as permittivity distributions, to structured outputs, such as electric field vectors (provided the domain is regular and well-represented in grid format), existing research works have focused on estimating and reconstructing permittivity from measured data [6]. The direct prediction of full electric potential distributions in the ECT numerical model using Graph Convolutional Networks remains underexplored.

In this proof-of-concept study, we demonstrate the feasibility of using Graph Convolutional Networks (GCNs) as fast surrogates for FEM-based forward modeling in Electrical Capacitance Tomography to directly predict all eight electric potential distributions for a circular ECT sensor with eight electrodes. The GCN is trained on synthetic data generated using FEM simulations under various permittivity configurations. Once trained, the network directly outputs the complete set of electric potential distributions corresponding to each excitation pattern. At this stage, our focus was on evaluating the method under controlled conditions using high-fidelity FEM simulations. It is important to note here that the present study focuses exclusively on the forward problem in ECT. Unlike inverse ML models that directly reconstruct permittivity, our approach predicts electric potential fields as a fast FEM surrogate. Such forward surrogates provide physically consistent fields from which capacitances and sensitivity matrices can be derived, offering flexibility for integration into different reconstruction pipelines. In this proof-of-concept work, we limit ourselves to demonstrating the feasibility of direct field prediction using GCNs.

Beyond demonstrating feasibility, such a model has several potential applications. First, it can serve as a fast surrogate for FEM within iterative reconstruction algorithms, substantially reducing computational costs. Second, it can be used to generate large synthetic datasets of physically consistent fields for training inverse problem solvers. Third, the predicted fields allow the derivation of capacitances and sensitivity matrices, making the model adaptable to different ECT processing pipelines. Finally, due to its low inference time, the approach may enable real-time or embedded ECT applications. In this work, we focus on a proof-of-concept validation, while these application scenarios are left for future development.

To evaluate the physical validity of the predicted fields, we compute inter-electrode capacitances. These results are compared against those obtained from FEM simulations. Our findings indicate that the GCN accurately approximates raw electric fields, enabling precise derivation of associated physical quantities. This paves the way for real-time forward modeling in ECT systems.

The primary contributions of this work are as follows:

- A novel application of Graph Convolutional Networks is proposed to directly and accurately predict complete electric potential distributions within an eight-electrode Electrical Capacitance Tomography sensor. This approach bypasses traditional computationally intensive Finite Element Method simulations for the forward problem.
- The model leverages the Spline Convolutional (SplineConv) operator, which is specifically designed to incorporate and utilize multi-dimensional edge attributes. This enables the model to exploit electrode-related proximity information embedded in the graph structure, leading to rich and physically informed representations from FEM-generated graph data.
- A rigorous evaluation of the prediction fidelity is conducted through comprehensive comparisons. The GCN-predicted electric potential fields are quantitatively assessed against reference FEM-generated fields. Furthermore, the accuracy of simulated capacitance values derived from the predicted potentials is validated against FEM-calculated capacitances.
- This work provides a compelling proof-of-concept demonstrating that GCN-based surrogate models can effectively replace conventional FEM solvers for achieving near real-time forward modeling in ECT. This significantly reduces the computational overhead associated with the forward problem, paving the way for faster iterative reconstruction algorithms or online monitoring applications.

## 2. Background

Electrical Capacitance Tomography is a non-invasive imaging technique that reconstructs permittivity distributions within a domain by measuring capacitance between electrode pairs [3]. Solving the forward problem by predicting capacitance measurements from known permittivity distributions is crucial for accurate image reconstruction [7,8,9].

Traditional methods, such as the Finite Element Method, offer high accuracy but are computationally intensive, limiting real-time applications [10,11]. Recent advancements have explored data-driven approaches to address these limitations [12].

Machine learning techniques, particularly deep learning, seem to be promising in approximating the forward problem efficiently. Various types of neural networks have been successfully applied to address this problem. For example, the application of feed-forward neural networks for solving the forward problem can be found in [13]. This solution can also be combined with an analysis of object features [14]. Deep learning has proven to be effective in related fields, such as impedance tomography. For example, CNNs were used in [15] and diffusion-based generative models in [16]. Conditional Generative Adversarial Networks (cGANs), foundational to many image-to-image translation applications, hold great potential in process tomography for reconstructing images from sensor data or enhancing their quality [17,18].

Convolutional Neural Networks are increasingly used as surrogate models (also known as metamodels or response surface models) to approximate complex, computationally expensive simulations or experiments. Their strength lies in handling high-dimensional input data, especially images or grid-like structures [19].

Graph Neural Networks, especially Graph Attention Networks (GATs), have emerged as powerful tools for modeling data on irregular domains, such as meshes used in FEM. Deep learning in inverse image reconstruction problems can be found, for example, in [20]. GATs, with their attention mechanisms, allow for dynamic weighting of neighboring nodes, enhancing the model’s ability to focus on relevant features [21].

Incorporating physical priors into machine learning models has also been a focus area. Features derived from the FEM stiffness matrix, such as mean, standard deviation, and variance, provide valuable information about local numerical behavior and have been used to enhance model performance [22,23]. Polish researchers have significantly contributed to the field of ECT. Kryszyn et al. developed the ECTsim toolbox for 3D modeling and image reconstruction in ECT [22]. Wanta et al. proposed a finite volume method using a quadtree non-uniform structured mesh for ECT modeling [23]. Ivanenko et al. explored supervised learning approaches for image reconstruction in wearable electrical impedance tomography systems [24]. Despite these advancements, challenges remain in achieving real-time, accurate forward modeling in ECT. The integration of GNNs, particularly GATs and GCNs, with physical priors derived from FEM offers a promising direction for future research.

## 3. Methodology

### 3.1. ECT Sensor Configuration and Forward Problem Definition

**Figure 1:**
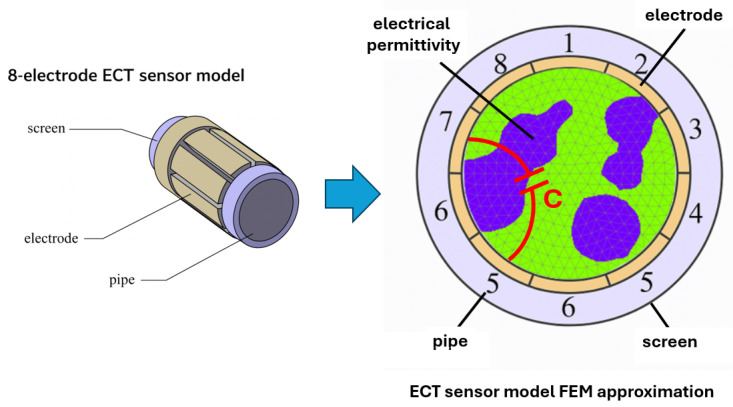
Eight-electrode ECT sensor model and its FEM approximation with key components.

In this research study, we consider a two-dimensional Electrical Capacitance Tomography setup consisting of eight electrodes uniformly distributed around a circular sensing domain—see Figure 1.

The forward problem in ECT involves computing the electric potential distribution $\phi_{k}\left(r\right)$ for each of the k=1,…,8 independent electrode excitation patterns. These potential distributions are essential for deriving mutual capacitances used in ECT image reconstruction.

The forward model in ECT model is governed by the quasi-static Maxwell equation in the following form:

(1) $$\nabla \cdot (\varepsilon \left(r\right)\nabla \phi_{k}\left(r\right))=0,\;r\in \Omega ,$$

where ε(r) is the spatially varying permittivity distribution, $\phi_{k}\left(r\right)$ is the electric potential for excitation pattern *k*, and Ω denotes the imaging domain. The boundary conditions are applied to simulate the excitation of each electrode while grounding others according to the excitation scheme. Dirichlet conditions are set for excited and grounded electrodes, and Neumann zero-flux conditions are applied on the insulating boundary.

For each pattern *k*, the resulting electric potential field $\phi_{k}\left(r\right)$ provides a complete description of the system’s electrostatic response, and the electric field can be derived as follows:

(2) $$E_{k}\left(r\right)=-\nabla \phi_{k}\left(r\right).$$

Mutual capacitance between electrodes *m* and *n* can then be calculated using the electric field-based energy formulation:

(3) $$C_{m,n}=\frac{1}{V^{2}}\int_{\Omega }\varepsilon \left(r\right)\nabla \phi_{m}\left(r\right)\cdot \nabla \phi_{n}\left(r\right),dr,$$

where *V* is the applied voltage (typically normalized to 1V in experimental conditions).

The total number of unique capacitance values for an *n*-electrode system is $\frac{n(n-1)}{2}$. For n=8, this yields 28 distinct independent values. These quantities are used both as reference outputs and as a means to evaluate the physical consistency of GCN-predicted potentials.

### 3.2. Dataset Generation Using FEM

To train, validate, and test our GCN-based surrogate model, we generated a dataset of 60,000 samples using the Finite Element Method. Each sample includes the following:

- A circular domain discretized into 335 nodes and 604 triangular elements.
- A random permittivity distribution composed of 1 to 5 separate or overlapping inclusions with varying shapes and contrasts (in the range 1–4 of relative electrical permittivity).
- FEM-computed electric potential values at each of the 335 mesh nodes for all eight excitation patterns.
- Eight additional features extracted from the row of the FEM system matrix corresponding to a given sample: the corresponding eigenvalue, as well as the row-wise mean, standard deviation, minimum, maximum, range (max–min), sum, and variance, which capture structural and spatial patterns related to the electrode configuration and mesh topology.

The full dataset comprised 60,000 randomly shuffled permittivity maps and their corresponding electric potentials, which were used as input–output pairs for training, validation, and testing of the graph neural network surrogate model. From this, an independent test set of 10,000 samples was separated, while the remaining 50,000 samples were split into training (40,000) and validation (10,000) subsets using an 80:20 ratio. In addition, a small subset of 30 samples was randomly selected from the test set to serve as illustrative examples for qualitative and quantitative analysis in the figures.

**Figure 2:**
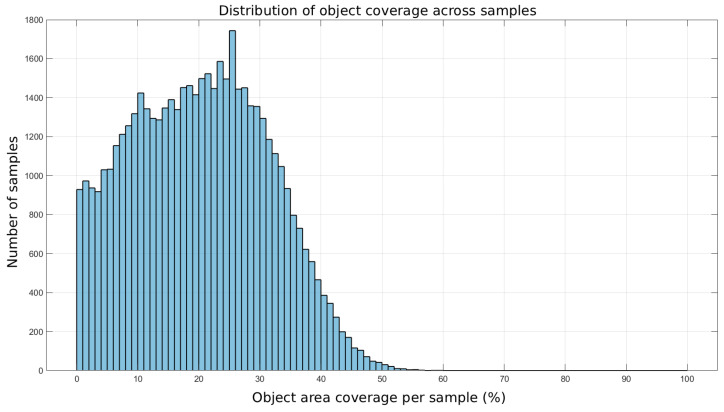
Histogram of permittivity inclusion coverage across the training dataset.

To better characterize the training dataset, we analyzed the distribution of permittivity inclusion coverage across all generated samples. Figure 2 shows a histogram of the percentage of the sensor area occupied by high-permittivity inclusions. The results confirm that the dataset spans a broad range of scenarios, from very sparse inclusions to cases where inclusions occupy nearly half of the sensing domain. The mean coverage across all samples is 20.29%, ensuring that the network is exposed to both low- and high-contrast configurations during training.

**Figure 3:**
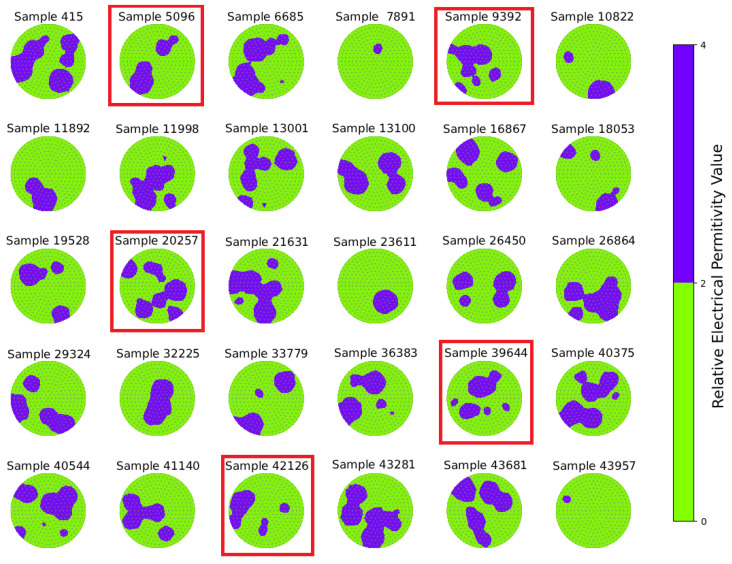
Permittivity distributions for 30 representative samples from the test set, visualized over the finite element mesh, chosen randomly from the 10,000 test samples set. Each subplot corresponds to one sample index.

Figure 3 presents 30 randomly selected permittivity distributions from the test set for subsequent qualitative and quantitative analysis. The distributions are visualized over the finite element mesh to illustrate the diversity and spatial complexity of the test geometries. From these illustrative cases, we selected five representative examples (red highlights) to present detailed error analyses (physical properties, pMAE, pMSE, cMAE, cMSE) in Section 4.

The FEM mesh used in this study contained 335 nodes and 604 triangular elements. This relatively coarse discretization was chosen to balance dataset generation cost and model training feasibility, while still capturing the essential features of potential distributions in a 2D circular sensor. As both FEM-based reference and GCN predictions were computed on the same mesh, the comparison remains consistent.

### 3.3. GCN-Based Graph Model Architecture for Electric Potential Prediction

#### 3.3.1. Input and Output Data Representation

We designed a Graph Convolutional Network that maps the permittivity distribution of a circular domain to a full set of electric potential values at the FEM mesh nodes. The model inputs a graph G=(V,E), which represents the FEM grid, where V is a set of vertices, and E⊆V×V is a set of edges.

Graph node features $v_{i}\in V$ represent physical or spatial attributes derived from FEM simulations for ECT reconstruction. These concatenated features include eigenvalues of the global stiffness matrix, vertex-wise material permittivity information, and statistical descriptors of the global stiffness matrix rows. Specifically, mean, standard deviation, minimum, maximum, range, sum, and variance of each node’s row in the stiffness matrix are used, providing localized insights into connectivity and the permittivity environment.

Edges $e_{ij}=\left(v_{i},v_{j}\right)\in E$ where i≠j connect neighboring nodes $v_{i}$ and $v_{j}$ in the FEM grid. Finally, edge attribute vectors $w_{ij}$ encode relative geometric information for spline kernels. Specifically, for each edge $e_{ij}$, each component of the vector $w_{ij}$ indicates a weighted proximity to a specific electrode. If we denote the position of node *i* as $p_{i}$, node *j* as $p_{j}$, and electrode *k* as $e_{k}$, then the *k*-th component of the edge attribute vector, $w_{ij,k}$, is calculated as follows:

(4) $$w_{ij,k}=\frac{1}{∥p_{i}-e_{k}∥+∥p_{j}-e_{k}∥+\epsilon }$$

where ∥·∥ denotes the Euclidean distance, and ϵ is a small constant added to prevent division by zero. This formulation ensures that edges geometrically closer to a particular electrode contribute more significantly to the convolutional operation related to that electrode’s influence.

The model outputs a 335×8 tensor, where each column represents the predicted nodal electric potentials corresponding to one of the 8 distinct electrode excitation patterns. This comprehensive output provides the full electric potential distributions across the sensing domain for various electrode configurations, directly reflecting the electric field generated as a consequence of the input permittivity distribution within the ECT system.

#### 3.3.2. Architecture Overview

The model is a Graph Convolutional Network designed to process graph-structured data representing FEM grids. Its architecture is built upon a sequence of Spline Convolutional layers (SplineConv), which are adept at learning from continuous edge attributes. Specifically, the SplineConv operator applies learnable continuous kernel functions parameterized by B-splines:

(5) $$x_{i}^{\left(l+1\right)}=\sum_{j\in N(i)}B\left(w_{ij}\right)W^{\left(l\right)}x_{j},$$

where $w_{ij}$ are edge attributes, B(w) is a B-spline basis function, and $W^{\left(l\right)}$ is the trainable weight matrix.

The proposed model comprises five SplineConv layers, forming an effective deep learning architecture. The input layer transforms the initial node features from 9 input channels to 32 channels. Then follows three hidden layers, which process the intermediate features, maintaining the 32 channels in dimension. The final fifth layer maps the refined hidden representations to the desired eight-channel output dimension, producing the model’s direct prediction for eight electrodes.

All SplineConv layers share common parameters. Each convolution aggregates neighborhood information using eight-dimensional B-spline kernels of size 3. This is followed by ReLU activation and dropout with a probability p=0.1 for regularization in the initial four convolutional layers. The dimensionality of B-spline kernels is explicitly set to the number of electrodes, and thus the edge’s attribute vector dimensionality, allowing the convolution to be uniquely defined by the relative position or influence of each electrode on that edge. The aggregation scheme for the convolutional layers is set to mean, implying that features from neighboring nodes are averaged after being weighted by SplineConv layer. Afterward, the model processes these node representations through a Multi-Layer Perceptron (MLP) head. This MLP head consists of a 64-dimensional linear layer, followed by ReLU activation and a dropout layer with a probability of p=0.1. Finally, a 64-dimensional output linear layer, along with a sigmoid activation function, produces the final predictions for electric potential values at all mesh nodes for each excitation pattern.

For FEM meshes with 335 nodes and eight excitation patterns, as considered in this study, the model outputs a tensor of shape 335×8.

**Figure 4:**
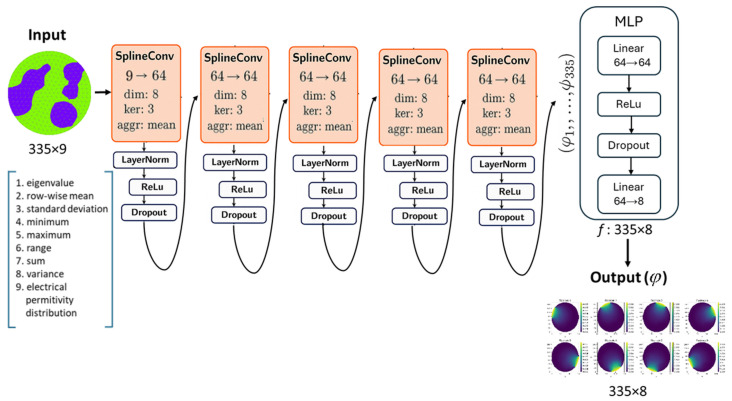
Block diagram of the proposed GCN model architecture. Nine features assigned to the FEM mesh-based graph node are processed through multiple SplineConv layers, normalized, activated, and passed through an MLP to predict eight FEM-mesh node-based distributions of electric potential for specific ECT electrode. The input is a simulation of the electrical permittivity distribution, where the blue color represents high-permittivity inclusions and the green color indicates the high-permittivity background. The output consists of eight predictions of the electrical potential, where bright yellow regions correspond to high electric potential and dark blue regions represent low electric potential.

The diagram that shows the flow of data through the proposed GCN model is presented in Figure 4.

#### 3.3.3. Training Setup and Parameters

The model was trained on a dedicated NVIDIA RTX A6000 GPU with 48 GB of RAM. For architectural settings as described in Section 3.3, the model contains approximately 28.05 million trainable parameters and consumes 1.7GB of Video RAM during operation.

Training was conducted for a maximum of 200 epochs, employing an AdamW optimizer with an initial learning rate of 0.003 and a weight decay parameter of 0.00001. To adapt the learning rate dynamically, a StepLR scheduler was utilized, which reduces the learning rate by a factor of 0.8 if both the validation mean squared error (MSE) does not show improvement for 30 consecutive epochs. The training process was performed with a batch size of 32.

The optimization objective was the Mean Squared Error (MSE), calculated with a sum reduction for the training loss. To prevent overfitting and enhance generalization, an early stopping mechanism was implemented with a patience of 30 epochs; training ceased if the validation MSE did not decrease for 30 consecutive epochs. The model’s parameters corresponding to the lowest validation MSE achieved during training were persistently saved, and the final state of the model’s parameters at the end of the training routine was also preserved.

**Figure 5:**
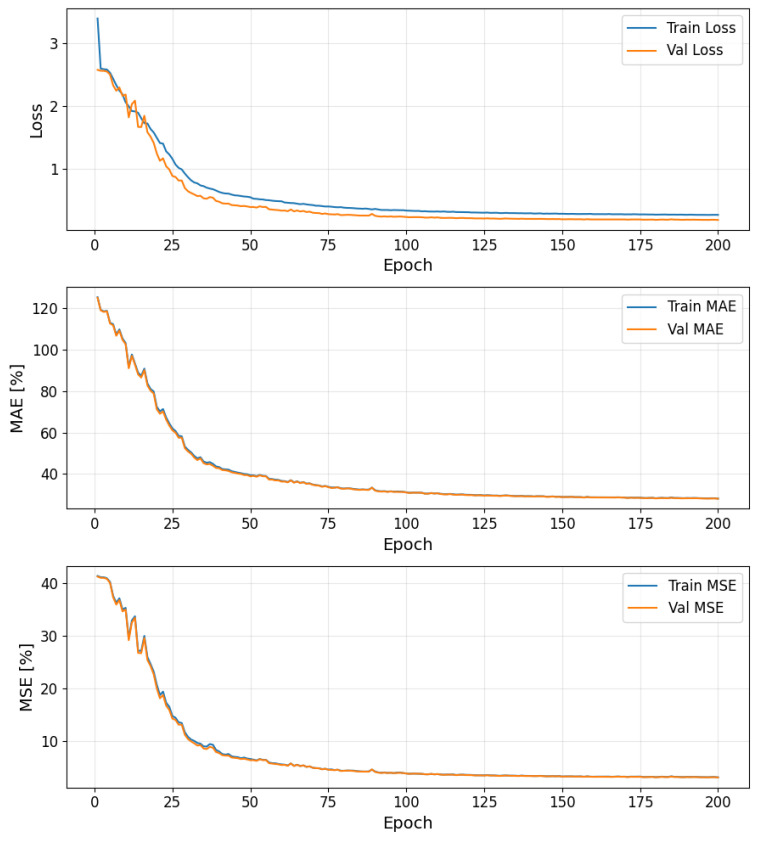
Training and validation loss, MSE and MAE curves during model training.

Figure 5 presents the training and validation curves for loss, mean absolute error (MAE), and mean squared error (MSE) over the course of training. It is worth noting that the loss curve (top panel) reflects the sum-reduced MSE used during optimization, while the MSE curve (bottom panel) reports the mean-reduced MSE per sample, hence the numerical discrepancy between them.

As shown in Figure 5, both training and validation losses, MAE, and MSE exhibit a consistent downward trend during the initial epochs, indicating effective learning. After approximately 100 epochs, all metrics begin to plateau, suggesting convergence. The close alignment between training and validation curves across all metrics implies that the model generalizes well without significant overfitting.

### 3.4. Post-Processing Using Predicted Potential: Capacitance Computation

From the predicted electric potentials, we computed physically relevant quantities to evaluate model fidelity. These post-processing steps were based on the ECT forward problem theory and are crucial for assessing the physical consistency of the surrogate model.

In practical implementation of Equation (3), the integral is discretized over each triangular element of the FEM mesh:

(6) $$C_{m,n}\approx \frac{1}{V^{2}}\sum_{e\in T}\varepsilon_{e}\cdot \nabla \phi_{m}^{e}\cdot \nabla \phi_{n}^{e}\cdot A_{e},$$

where T is the set of triangular elements, $\varepsilon_{e}$ is the permittivity in element *e*, $\nabla \phi_{k}^{e}$ is the gradient of the potential in that element (assumed constant per element), and $A_{e}$ is the element area.

These post-processing capacitance computations not only validate the accuracy of potential predictions but also assess whether the learned model retains the physical interpretability required for integration into ECT forward and inverse pipelines.

### 3.5. Evaluation Metrics

To assess model performance of our GCN-based surrogate model, we applied the following metrics:

- Qualitative visualization of predicted vs. reference electric potential distributions.
- Percentage MSE (pMSE) and MAE (pMAE) between CNN-based and FEM-based electric potential distributions over all nodes defined as follows:
  (7) $$\mathrm{pMSE}=\frac{1}{N}\sum_{i=1}^{N}{\left(\phi_{\mathrm{CNN}}\left(i\right)-\phi_{\mathrm{FEM}}\left(i\right)\right)}^{2}*100$$
  (8) $$\mathrm{pMAE}=\frac{1}{N}\sum_{i=1}^{N}\left|\phi_{\mathrm{CNN}}\left(i\right)-\phi_{\mathrm{FEM}}\left(i\right)\right|$$
- Capacitance Error Metrics, specifically cMSE and cMAE given by Equation (10) and Equation (9), respectively, that evaluate the accuracy of the predicted capacitance values against the reference.
  (9) $$\mathrm{cMAE}=\frac{1}{N}\sum_{i=1}^{N}\left|C_{\mathrm{pred},i}-C_{\mathrm{true},i}\right|$$
  (10) $$\mathrm{cMSE}=\frac{1}{N}\sum_{i=1}^{N}{\left(C_{\mathrm{pred},i}-C_{\mathrm{true},i}\right)}^{2}$$

Formulas (7)–(10) follow the standard definitions of mean squared error (MSE) and mean absolute error (MAE). We chose to write them separately for potentials (pMSE, pMAE) and capacitances (cMSE, cMAE) to highlight their distinct roles and physical units: potential errors are expressed in volts, while capacitance errors are expressed in picofarads. The prefix *p* denotes that the metric is calculated on potential distributions, whereas *c* indicates capacitance-domain errors. Only pMSE is expressed as a percentage, since electric potentials are normalized to the [0–1 V] range, which makes relative errors directly interpretable. In contrast, capacitances span a meaningful physical range, where absolute deviations in pF are more informative than percentage scaling. Throughout this work, the reference corresponds to values obtained from high-fidelity FEM simulations, which provide the benchmark against which GCN predictions are compared.

## 4. Results

This section presents the results of evaluating the GCN-based surrogate model for predicting electric potential distributions in an eight-electrode circular ECT sensor. The model’s performance was assessed on an independent test set of 10,000 samples, considering both its ability to approximate FEM-generated electric potentials directly and its capacity to produce accurate derived quantities such as capacitance matrices. To provide both a statistically sound assessment and qualitative insight, we adopted a two-level evaluation strategy. First, we report global statistical metrics (mean, standard deviation, minimum, and maximum of MAE and MSE) across the entire test set to quantify the model’s overall accuracy and robustness. Second, we present five representative test samples (‘highlights’) that illustrate diverse cases and allow for detailed error analysis, including physical properties as well as pMSE, pMAE, cMSE, and cMAE values. This combined approach provides both a comprehensive statistical evaluation and an in-depth demonstration of the model’s behavior on specific test cases.

### 4.1. Global Statistical Evaluation

**Figure 6:**
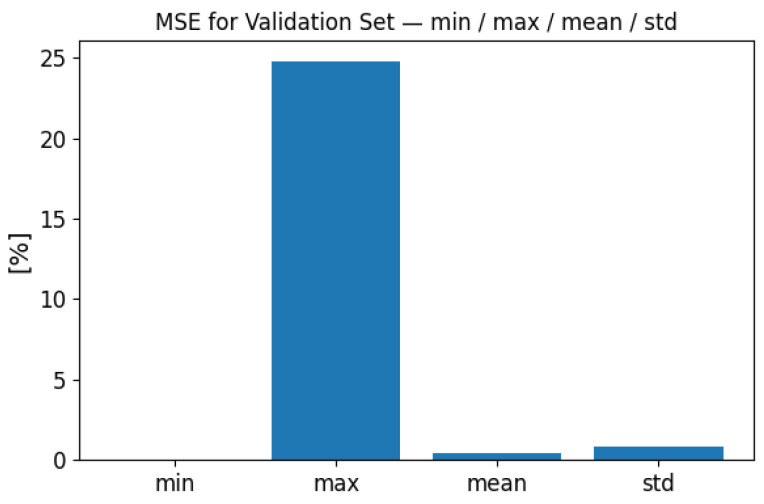
Global MSE performance evaluation on the full test set (10,000 samples). Summary statistics of MSE are shown: minimum, maximum, mean, and standard deviation.

**Figure 7:**
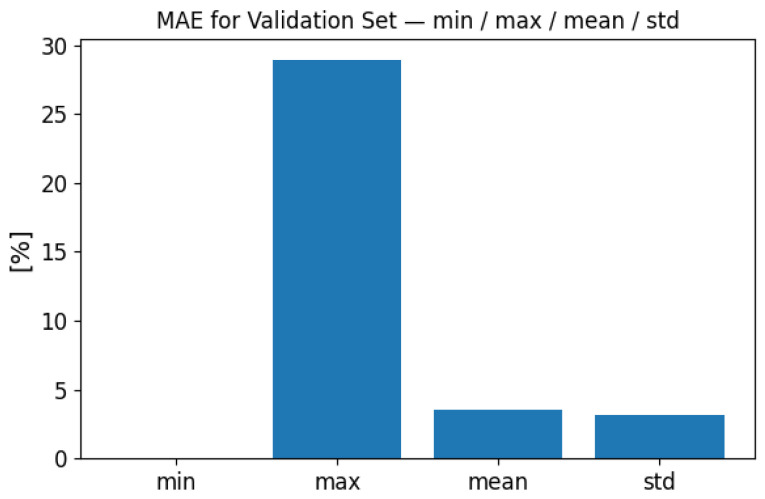
Global MAE performance evaluation on the full test set (10,000 samples). Summary statistics of MAE are shown: minimum, maximum, mean, and standard deviation.

To demonstrate the model’s performance, we carried out a global statistical evaluation over the entire independent test set of 10,000 samples. Figure 6 and Figure 7 present summary statistics of the global MSE and MAE values. For the test set, the global MSE ranged from less than 0.000001% up to 24.81%, with a mean of 0.40% and a standard deviation of 0.85%. The global MAE ranged from 0.001% to 28.96%, with a mean of 3.51% and a standard deviation of 3.16%. These results demonstrate that while a few cases exhibit higher error, the overall performance remains stable across the full test distribution.

### 4.2. Electric Potential Prediction Accuracy

**Figure 8:**
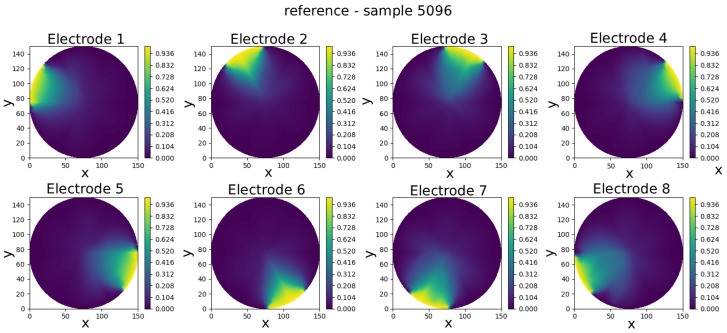

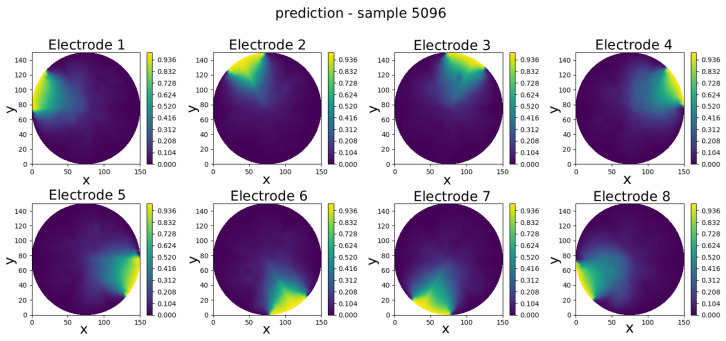
Sample 5096—comparison of reference and predicted electric potential distributions for each electrode in an eight-electrode ECT numerical sensor model.

**Figure 9:**
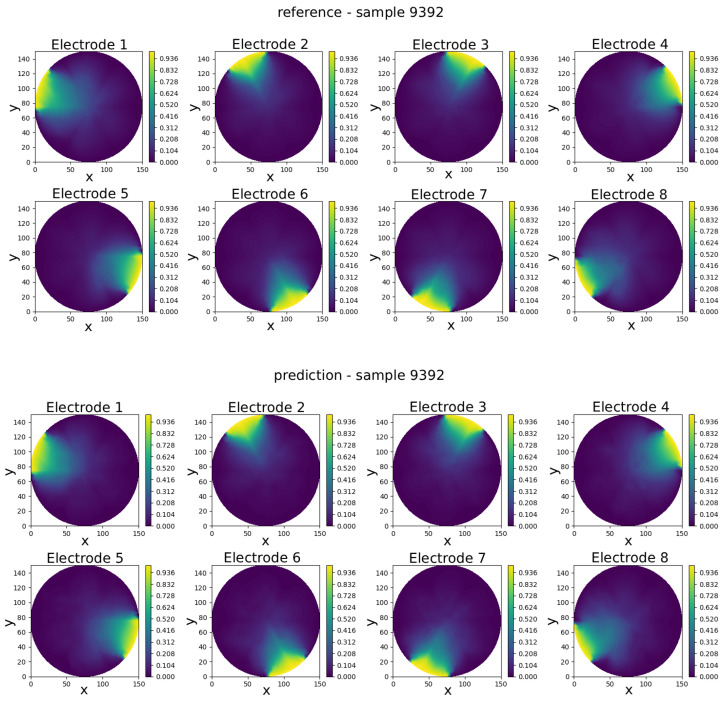
Sample 9392—comparison of reference and predicted electric potential distributions for each electrode in an eight-electrode ECT numerical sensor model.

**Figure 10:**
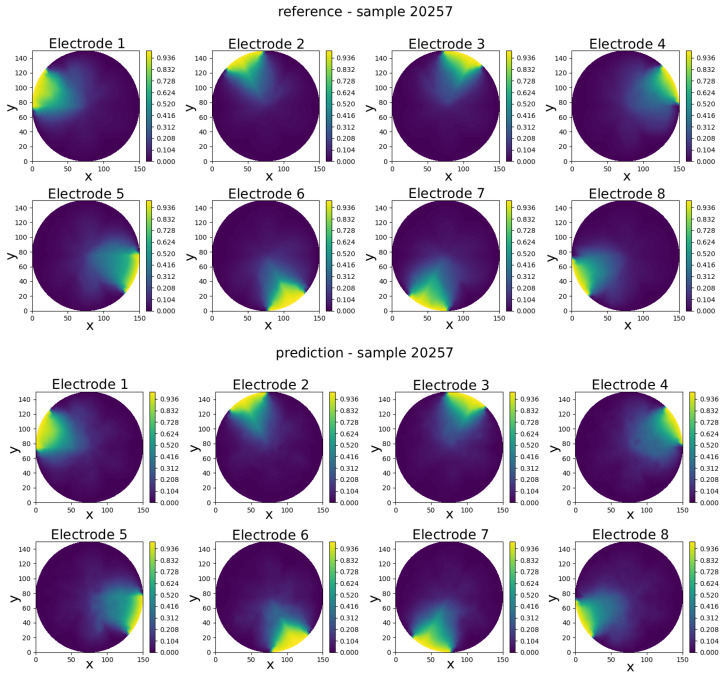
Sample 20257—comparison of reference and predicted electric potential distributions for each electrode in an eight-electrode ECT numerical sensor model.

**Figure 11:**
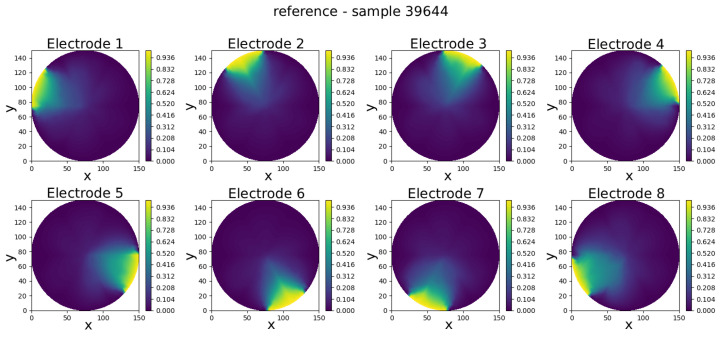

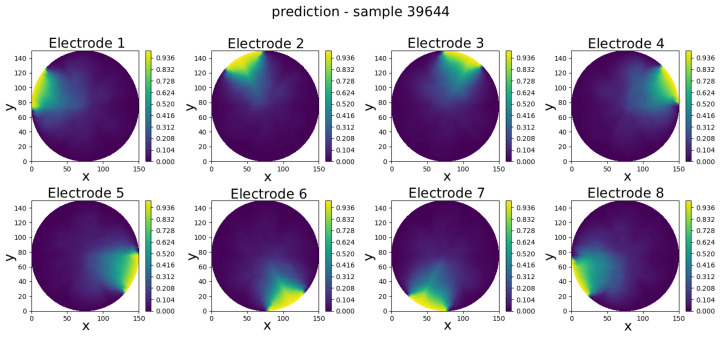
Sample 39644—comparison of reference and predicted electric potential distributions for each electrode in an eight-electrode ECT numerical sensor model.

**Figure 12:**
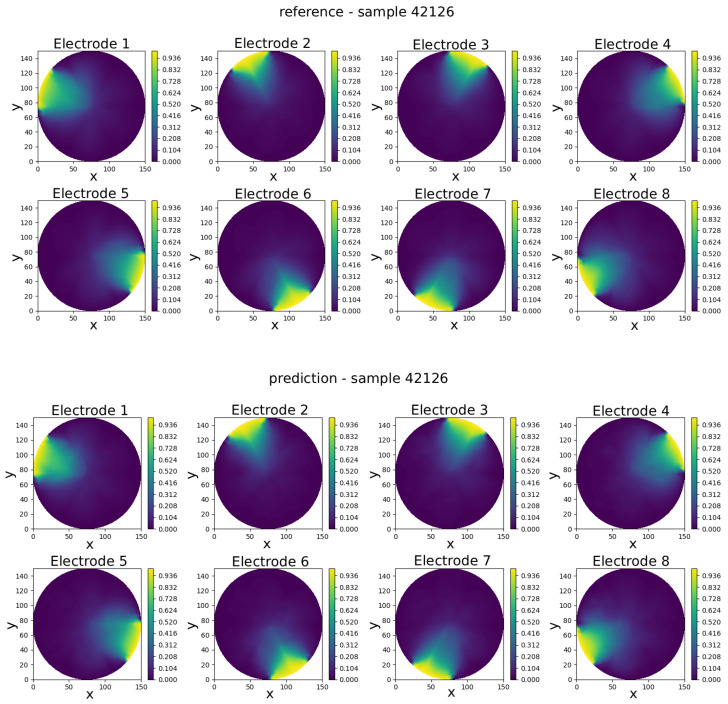
Sample 42126—comparison of reference and predicted electric potential distributions for each electrode in an eight-electrode ECT numerical sensor model.

The GCN neural model was trained to predict eight full-field electric potential distributions, each represented by scalar values at the 335 FEM mesh nodes of a circular domain. The comparison between real and predicted electric potential distributions across each electrode in an eight-electrode ECT numerical sensor model is visually presented in Figure 8, Figure 9, Figure 10, Figure 11 and Figure 12. Within each figure, the top panel displays the reference results, while the GCN’s prediction is shown in the bottom panel.

Visual inspection of results shows that the predicted potentials closely resemble their FEM counterparts, capturing key features such as field symmetry and boundary alignment.

**Figure 13:**
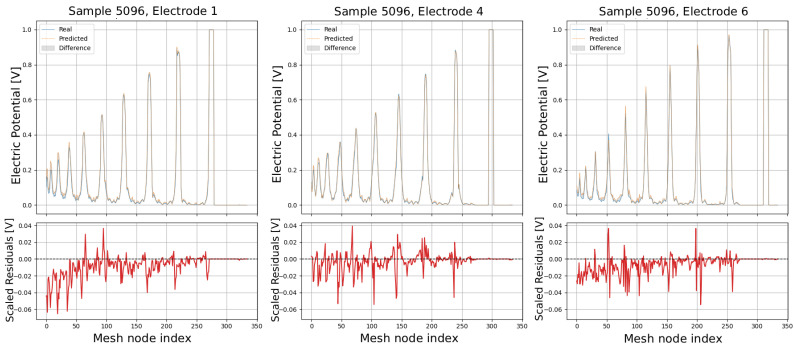
Comparison of predicted and real electric potential distributions for electrodes 1, 4, and 6 in Sample 5096. **Top row**: FEM reference potentials (blue) vs. GCN predictions (orange), with shaded areas showing the local differences. **Bottom row**: residuals (FEM–GCN), demonstrating small deviations centered around zero.

**Figure 14:**
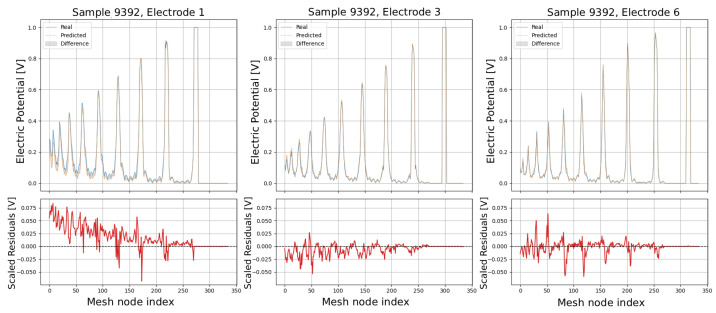
Comparison of predicted and real electric potential distributions for electrodes 1, 4, and 6 in Sample 9392. **Top row**: FEM reference potentials (blue) vs. GCN predictions (orange), with shaded areas showing the local differences. **Bottom row**: residuals (FEM–GCN), demonstrating small deviations centered around zero.

**Figure 15:**
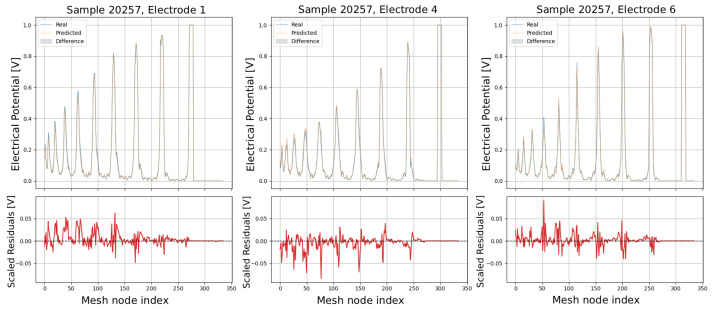
Comparison of predicted and real electric potential distributions for electrodes 1, 4, and 6 in Sample 20257. **Top row**: FEM reference potentials (blue) vs. GCN predictions (orange), with shaded areas showing the local differences. **Bottom row**: residuals (FEM–GCN), demonstrating small deviations centered around zero.

**Figure 16:**
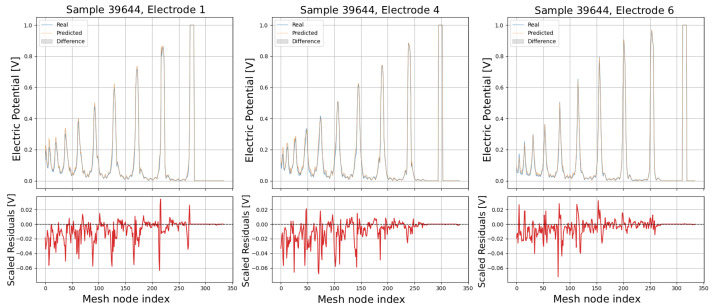
Comparison of predicted and real electric potential distributions for electrodes 1, 4, and 6 in Sample 39644. **Top row**: FEM reference potentials (blue) vs. GCN predictions (orange), with shaded areas showing the local differences. **Bottom row**: residuals (FEM–GCN), demonstrating small deviations centered around zero.

**Figure 17:**
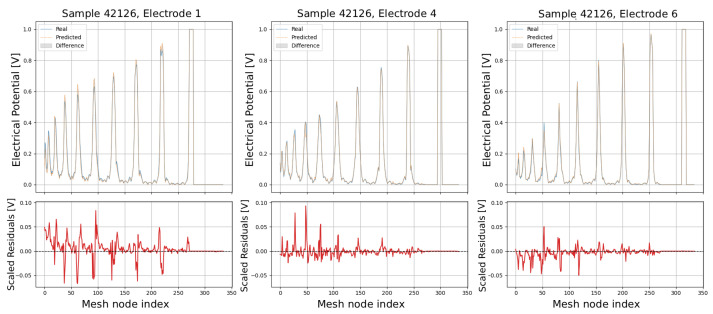
Comparison of predicted and real electric potential distributions for electrodes 1, 4, and 6 in Sample 42126. **Top row**: FEM reference potentials (blue) vs. GCN predictions (orange), with shaded areas showing the local differences. **Bottom row**: residuals (FEM–GCN), demonstrating small deviations centered around zero.

The following plots (see Figure 13, Figure 14, Figure 15, Figure 16 and Figure 17) show a comparison between predicted and real electric potential value distributions over mesh nodes for electrodes 1, 4, and 6, across multiple representative samples.

In the upper panels, the predicted curves (orange) align very closely with the real curves (blue), with only minor deviations visible at peak regions and around sharp transitions. The shaded areas highlight the local differences, which remain small relative to the overall signal amplitude. The lower residual plots (red curves) confirm this, showing that most residuals fluctuate around zero and generally stay within ±0.05 V, indicating high predictive accuracy. While small discrepancies appear at certain peak positions, especially in samples with higher potential amplitudes, the error magnitudes remain low and consistent across electrodes, demonstrating strong agreement between the predicted and real distributions.

Quantitatively, the mean squared error (pMSE) and mean absolute error (pMAE) between the CGN and FEM electric potentials were also computed for five selected test samples (’highlights’) to illustrate the diversity of model behavior under different physical conditions. These examples of MSE and MAE errors are reported in Table 1.

The results in Table 1 show that the model achieves consistently low prediction errors across the five selected samples. The mean absolute error (pMAE) ranges from 0.0188 V (Sample 42126) to 0.0277 V (Sample 9392) according to the voltage range from 0 to 1 V, indicating that voltage predictions are highly accurate and stable. Correspondingly, the mean squared error (pMSE) values remain below 0.12%, with the lowest error observed for Sample 42126 (0.057%) and the highest for Sample 20257 (0.120%). The slight increase in error for some samples suggests that more complex permittivity distributions are somewhat harder to predict, but the overall performance confirms the model’s robustness and reliability across varying test cases.

### 4.3. Capacitance Comparison

Capacitance matrices were computed using the predicted electric potentials and compared with those derived from FEM simulations according to Equation (6). The results indicate medium to high correlation between the two electric field approximation methods. Table 2 presents cMSE and cMAE values computed for the selected samples.

The error analysis confirms the model’s acceptable overall performance. Among the selected illustrative examples, Sample 9392 shows excellent agreement between predicted and real values (cMSE = 0.78%, cMAE = 0.73 pF), while Sample 42126 also achieves a low error (cMSE = 5.96%, cMAE = 1.97 pF), indicating reliable predictions in these instances. In contrast, Samples 20257 and 39644 exhibit noticeably higher errors, particularly Sample 39644 (cMSE = 65.92%, cMAE = 5.41 pF), suggesting difficulties in reproducing larger capacitance variations or sharp peaks. Sample 5096 demonstrates moderate errors (cMSE = 15.13%, cMAE = 2.74 pF), reflecting reasonably accurate tracking with some deviations.

**Figure 18:**
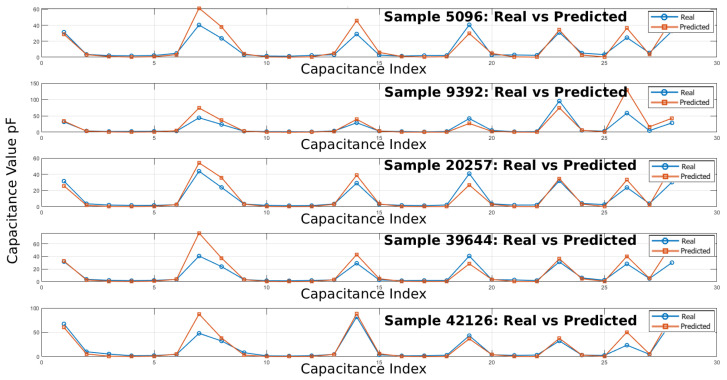
Real vs. predicted absolute capacitance values in pF for representative samples [5096, 9392, 20257, 39644, 42126].

Figure 18 shows the FEM- and GCN-predicted electric field-derived capacitance matrices for a test case, as well as their absolute difference matrix.

The comparison between real and predicted capacitance values across the selected samples demonstrates that the predictive model effectively captures the overall trends and shapes of the capacitance profiles. In smooth, low-variation regions, the predicted curves closely overlap with the real measurements, indicating strong baseline accuracy. Peaks and sudden fluctuations are generally well identified, though some discrepancies remain in the amplitude of sharp peaks and in tracking smaller oscillations. Overall, the predicted solution shows robust performance, with most deviations arising from underestimation or overestimation at high-variance points, suggesting that further refinement could focus on improving accuracy during rapid capacitance changes.

**Figure 19:**
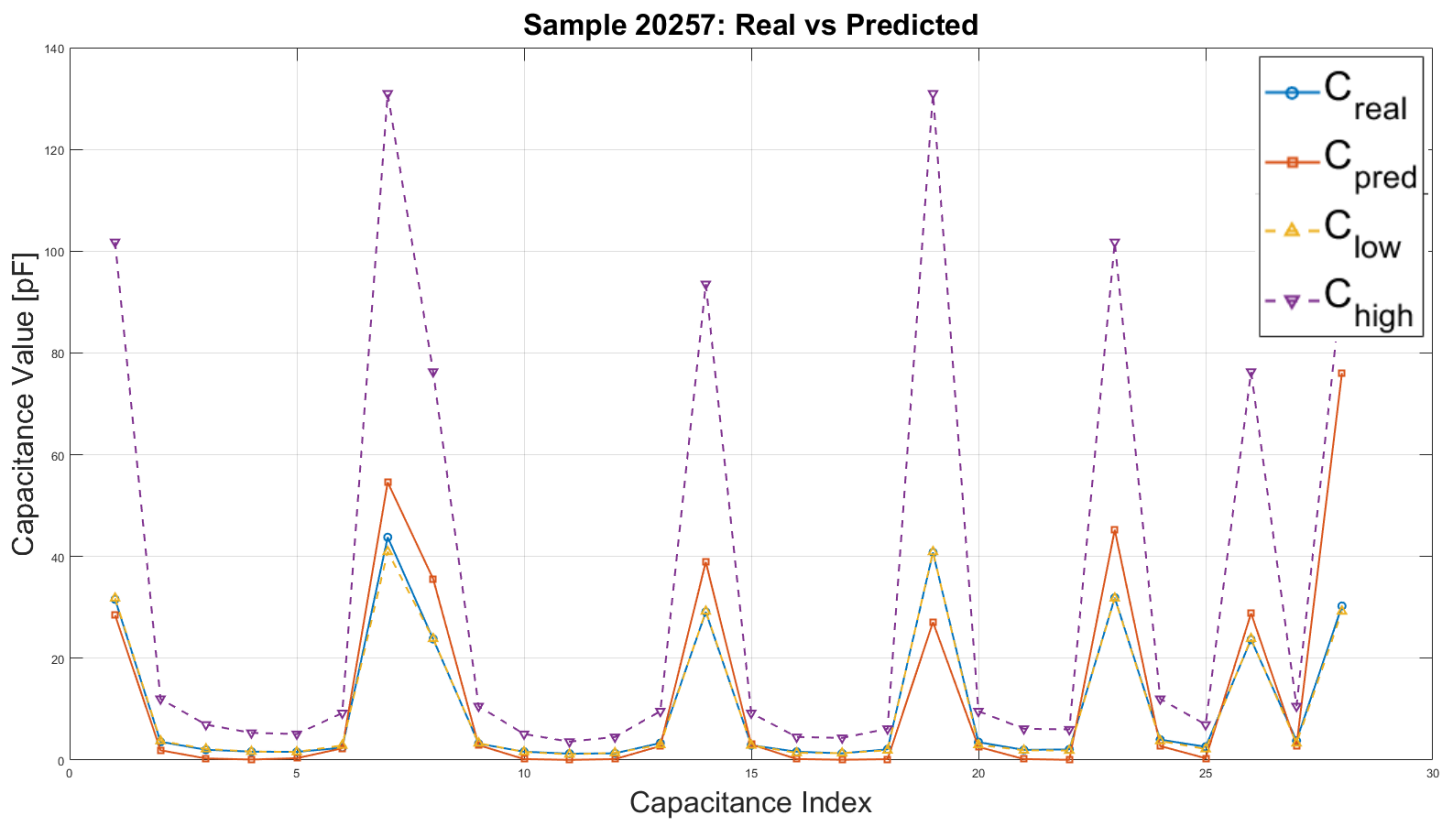
Distribution of absolute capacitance values in pF for an empty sensor, a sensor fully filled, and a representative training sample no. 20257.

To further characterize the dataset, Figure 19 presents the distribution of absolute capacitance values obtained from FEM simulations for three representative cases: (i) an empty sensor, (ii) a sensor fully filled with permittivity, and (iii) a typical example (sample 20257) from the training set. The results illustrate the expected range of capacitances and confirm that the simulations are physically consistent across different permittivity configurations.

### 4.4. Computation Time and Inference Speed

Execution times were compared between the proposed GCN surrogate and a conventional FEM solver. The FEM simulations were executed on a CPU, while neural network inference was performed on a GPU, reflecting the typical computing environments for each method. This comparison should therefore be viewed not as a strict device-to-device benchmark, but as a practical illustration of the reduced inference cost achievable with a trained model. On average, the GCN required only milliseconds per sample, yielding a several-fold reduction in computation time relative to FEM under these conditions. While the exact speedup factor naturally depends on hardware and implementation details, the results demonstrate the potential of GCN-based surrogates to substantially accelerate forward modeling. A more rigorous, device-normalized benchmark remains a subject for future work. Training the proposed GCN required approximately 800 s per epoch on an NVIDIA RTX A6000 GPU, with convergence typically reached within 100–150 epochs. As training is a one-time offline cost, the efficiency of the trained model is most relevant for deployment. The inference time for the GCN was approximately 7 milliseconds per full sample (i.e., all eight electric potential fields), compared to 30 milliseconds per sample for a standard FEM solver running on a CPU (Intel Core i7-9750H at 2.6 GHz). Under these conditions, this corresponds to a speedup of over fourfold.

## 5. Discussion

### 5.1. Model Performance

The results demonstrate that graph Convolutional Neural Networks (GCNs) can accurately predict electric potential distributions in a circular eight-electrode ECT sensor using a surrogate modeling approach. We selected the eight-electrode configuration because in our opinion and due to our experience, it is the most widely used benchmark in ECT research and provides a well-established test case. However this approach is not fundamentally limited to this setup and can be adapted to different electrode configurations. The strong correlation between GCN and FEM outputs across all eight excitation patterns confirms that GCNs can capture the underlying physics of the problem, even without explicit enforcement of physical laws.

Qualitative comparisons of FEM reference distributions and GCN predictions (Figure 4, Figure 5, Figure 6, Figure 7 and Figure 8) show that the model effectively reproduces the global spatial characteristics of the potential fields. In particular, the GCN closely follows FEM outputs in high-intensity regions near active electrodes, successfully capturing steep potential gradients and maintaining the circular symmetry imposed by the geometry. However, slight discrepancies arise in low-potential zones, where the model tends to smooth spatial variations. This leads to a minor underestimation of localized peaks and reduced detail in regions with gradual potential transitions, suggesting a tendency toward over-regularization. Despite these localized deviations, the overall magnitude range and spatial alignment remain highly consistent with FEM results. These findings indicate that the GCN generalizes well across electrode configurations while significantly reducing computational cost without compromising physical accuracy.

### 5.2. Error Analysis

Error analysis further supports the robustness of the approach. The global statistics evaluation across the entire independent test set of 10,000 samples confirms stable performance of the developed model. The absence of systematic error trends across five test samples suggests stable performance throughout the dataset. The consistently low pMSE and pMAE values indicate that the model has effectively learned the mapping from input permittivity distributions to corresponding output fields. Several factors likely contribute to this success: the use of numerically simulated data with limited noise, the relatively simple eight-electrode setup that produces smoothly varying spatial distributions, and the alignment of the GCN architecture with the spatial characteristics of the problem.

A detailed analysis indicates that although the overall performance is robust, a small number of samples in the test set exhibit higher error rates. A detailed analysis reveals that these are typically cases with highly heterogeneous permittivity distributions, which create sharp field gradients and localized peaks in the electric potential. These conditions are inherently more challenging for data-driven models, as even minor prediction errors in the potential can be amplified, leading to significant discrepancies in derived quantities like capacitance. It is important to note that these high-error samples are outliers and do not represent a systemic failure of the model, as evidenced by the consistently low errors across the vast majority of the dataset. This observation further highlights the model’s general reliability and provides valuable insight into its limitations, suggesting avenues for future research focused on handling such complex boundary cases.

While low error values are encouraging, they may also reflect the controlled nature of the dataset. Future evaluations on experimental or noisy data, as well as more complex two-dimensional ECT models, will be essential to assess robustness under real-world conditions.

It is also worth noting that the error metrics were deliberately kept separate for potentials and capacitances to preserve clarity of interpretation. The p-prefix consistently refers to potential-based errors, independent of units, while the percentage form was applied only to pMSE due to the normalized [0–1 V] voltage range. For capacitances, FEM-derived values served as the reference, and reporting errors in absolute units (pF) provides a direct measure of practical accuracy.

### 5.3. Computational Efficiency

An important advantage of the proposed framework is the substantial computational speed-up achieved without compromising reconstruction quality. This benefit is expected to increase with more complex ECT configurations, such as finer FEM meshes or three-dimensional electrode arrangements, where conventional solvers become increasingly burdensome. The reduced inference latency of the GCN enables real-time or near-real-time applications, allowing continuous industrial process monitoring and rapid detection of transient phenomena. In industrial ECT systems, this capability translates into processing hundreds of image reconstructions per second and integrating seamlessly with online control systems and feedback loops, facilitating advanced process control and adaptive sensing.

### 5.4. Limitations and Future Work

Despite the promising results, some limitations remain. The current model is trained exclusively on synthetic data and may face challenges when applied to real measurements that include noise or non-ideal electrode geometries. Furthermore, the architecture is specific to the tested sensor configuration and would require retraining for different geometries or electrode numbers. Future work may explore domain adaptation, physics-informed loss functions, or integration with inverse solvers to enhance generalization.

Another limitation relates to the range of architectural and training choices explored. In this proof-of-concept study, we focused on a single, well-established configuration of activation functions and optimizer to demonstrate feasibility. Sensitivity to architectural choices deserves more systematic investigation. In particular, alternative activation functions and optimizers could potentially influence convergence and prediction accuracy. While such an ablation study would be valuable, it lies beyond the scope of the present work due to the significant computational resources required for retraining large-scale models. We therefore highlight this as an important direction for future research, especially when extending the framework to more complex ECT electrode geometries and experimental datasets.

In the present study, we focused on Graph Convolutional Networks for direct prediction of electric potential distributions in ECT forward modeling. To the best of our knowledge, no prior published machine learning approaches have addressed this specific task. Existing ECT studies have concentrated primarily on the inverse problem of permittivity reconstruction, where CNNs, GANs, or MLPs have been applied. In other areas, machine learning surrogates for forward modeling have been explored on grid-based representations, but these methods are not readily transferable to the irregular FEM meshes characteristic of ECT. The graph-based representation adopted here is particularly suited to incorporating both mesh topology and electrode-related proximity information, offering a natural advantage over conventional grid-based architectures. While one could, in principle, adapt CNNs, MLPs, or physics-informed neural networks as baseline comparators, such adaptations would require nontrivial modifications to handle irregular meshes and may not fully capture the underlying physics. For this proof-of-concept study, we therefore prioritized demonstrating the feasibility of a graph-based approach. Systematic evaluation of GCNs alongside these alternative architectures nevertheless represents an important direction for future research.

### 5.5. Key Contributing Factors

Overall, the success of this method can be attributed to three key factors:

1. Structured input representations, where permittivity maps allow GCNs to effectively capture spatial relationships and field gradients.
2. Access to a rich FEM-generated training dataset, which enables the network to generalize well to unseen configurations.
3. Validation through derived physical quantities, such as inter-electrode capacitances, confirming the physical consistency of the predictions.

## 6. Conclusions

This work presented a proof-of-concept study demonstrating the feasibility of using graph Convolutional Neural Networks (GCNs) to directly predict electric potential distributions in Electrical Capacitance Tomography (ECT) systems. Evaluated on a circular eight-electrode configuration and validated against high-fidelity Finite Element Method (FEM) simulations, the proposed model showed strong agreement in both direct electric potential predictions and derived capacitance matrices.

The GCN achieved substantial computational efficiency, reducing inference time from tens of milliseconds to milliseconds per sample, while maintaining high physical accuracy. This near real-time capability makes the approach highly suitable for embedded and high-speed ECT applications, enabling continuous monitoring and rapid detection of transient phenomena that conventional FEM solvers would struggle to capture.

This study also demonstrated that a neural network trained solely on permittivity-to-potential mappings can generate physically consistent outputs, supported by validation through both direct field predictions and derived physical metrics. These findings establish a strong foundation for integrating deep learning surrogates into forward ECT modeling, offering a scalable and efficient alternative to traditional numerical solvers.

Despite the promising results, some limitations remain. The current model was trained exclusively on synthetic data and tailored to a specific sensor configuration, raising challenges in generalization to experimental measurements, noisy conditions, or alternative geometries. Future work should focus on extending the framework to handle diverse electrode arrangements, incorporating noise robustness, and exploring domain adaptation or physics-informed loss functions. The integration of GCN-based surrogates with inverse solvers and their extension to three-dimensional ECT configurations represent particularly promising directions.

Overall, this study highlights the viability of physics-informed deep learning in ECT, demonstrating that GCN-based surrogates can deliver accurate, efficient, and scalable alternatives to FEM for real-time industrial imaging applications.

## Figures and Tables

**Table 1:** Potential prediction Mean Squared Error (pMSE, expressed in percent) and Mean Absolute Error (pMAE, expressed in volts) for selected 5 test samples from an eight-electrode Electrical Capacitance Tomography numerical sensor model.

Sample Index	pMSE [%]	pMAE [V]
5096	0.110843	0.024506
9392	0.098940	0.027721
20257	0.119838	0.026305
39644	0.089179	0.019808
42126	0.056943	0.018794

**Table 2:** cMSE and cMAE of real vs. predicted capacitance values according to expected theoretical capacitance range $\tilde{0}$ pF–120 pF.

Sample	cMSE [%]	cMAE [pF]
5096	15.1324	2.7429
9392	0.7782	0.7261
20257	47.9526	5.0307
39644	65.9216	5.4097
42126	5.9615	1.9664

## Data Availability

The original contributions presented in this study are included in the article. Further inquiries can be directed to the corresponding author.

## Abbreviations

The following abbreviations are used in this manuscript:

ECT	Electrical Capacitance Tomography
FEM	Finite Element Method
GCN	Graph Convolutional Neural Network
MSE	Mean Squared Error
MAE	Mean Absolute Error
GPU	Graphics Processing Unit
CPU	Central Processing Unit
ReLU	Rectified Linear Unit
